# Family Support and Sociocultural Factors on Depression among Black and Latinx Sexual Minority Men

**DOI:** 10.3390/ijerph18136759

**Published:** 2021-06-23

**Authors:** Donte T. Boyd, S. Raquel Ramos, Camille R. Quinn, Kristian V. Jones, Leo Wilton, LaRon E. Nelson

**Affiliations:** 1 College of Social Work, Ohio State University, Columbus, OH 43210, USA; Quinn.395@osu.edu; 2 Center for Interdisciplinary Research on AIDS, New Haven, CT 06510, USA; laron.nelson@yale.edu; 3 Department, Rory Meyers College of Nursing, New York University, New York, NY 10010, USA; Raquel.ramos@nyu.edu; 4 School of Social Work, University of Texas at Austin, Austin, TX 78712, USA; kjones025@utexas.edu; 5 Department of Human Development, State University of New York at Binghamton, Binghamton, NY 13902, USA; lwilton@binghamton.edu; 6 Faculty of Humanities, University of Johannesburg, Johannesburg 2006, South Africa; 7 School of Nursing, Yale University, New Haven, CT 06477, USA; 8 St. Michael’s Hospital, Li Ka Shing Knowledge Institute, MAP Centre for Urban Health Solutions, Toronto, ON M5B 1W8, Canada

**Keywords:** families, depression, sexual minority men, stressful life events, internalized homophobia

## Abstract

Family-based approaches are critical for improving health outcomes in sexual minority men (SMM) of color. Yet, it is unclear how family context, internalized homophobia, and stress influence mental health outcomes among sexual minority men of color. From a cross-sectional sample of 448 participants, aged 16–24 years, survey data were analyzed to examine rates of family social support, the perception of sexuality by family, the stressfulness of life events, internalized homophobia, and other contextual variables on depression using linear regression. Our results indicated that an 86% increase in family social support was related to a −0.14 decrease in depression (ß = −0.14, *p* = 0.004). In addition, SMM who were separated by family and friends because of their sexuality were statistically significant and positively associated with depression (ß = 0.09, *p* < 0.001). Findings from our study suggest that the influence from the microsystem is salient in modifying mental health outcomes for SMM of color.

## 1. Introduction

Approximately 3.9 million persons in the United States identify as sexual minority men [1] (SMM). The term sexual minority refers to a person whose sexual identity is not exclusively heterosexual [2]. Due to the many identities SMM may hold, their lived experiences cannot be viewed in isolation. The intersectionality framework acknowledges the many facets of an individual’s identity and how these identities contribute to interlocking systems of power or inequality [3,4,5]. For example, SMM may have male gender privilege; however, when gender is intersected with race, ethnicity, and sexual identity, the context of one’s power status may shift depending on the context of their environment. Recent literature indicates that SMM are three times as likely to experience depression [6,7] and twice as likely to experience certain mental health disorders when compared to heterosexual men and women in the United States [7,8], thus highlighting how their sexual minority identity can impact their mental health and well-being.

According to previous research, major depression is common in SMM [9] and associated with a high disease burden [10]. Depression can be devastating to an individual’s quality of life and can decrease their productivity, physical health [11,12], and potentially increase their HIV risk [13]. In addition, depression has been associated with chronic comorbid conditions, such as cardiovascular disease [14], and an increase in alcohol use, substance misuse, and family disruption/rejection [12,15]. In sexual minority communities, family factors around sexual identity have a salient role. We define family factors broadly as family support, family values, and familial embarrassment that are portrayed by parents, caregivers, and/or sibling(s) from one’s biological or chosen family [16,17].

### 1.1. Family Factors

One family factor or dynamic that has been shown to have a deleterious effect on the mental health of SMM is rejection based on sexual identity [18,19]. Family disruption due to rejection has severe consequences for the physical and mental health of young adult SMM [18,19,20]. SMM young adults who have experienced family disruption because of rejection were found more likely to be suicidal and to have high levels of depression [20]. Conversely, we also know that the presence of parental support and positive communication has decreased HIV risk in Black sexual minority young people and adults [16,17]. Family factors are an exponentially significant factor in mitigating HIV risk and familial stigma and depression about sexual identity.

### 1.2. Family Social Support

Family social support has been associated as a protective factor among SMM [12]. Family social support includes both emotional as well as tangible support, specifically financial assistance and other resources [12]. The extant literature on family support has shown family members’ acceptance or rejection of one’s choice in sexual partners is associated with depression among SMM [12,21,22,23]. There is empirical evidence that highlights the nuance within family support; for instance, qualitative research highlighted that those who were rejected by some family members often found support from their mothers [24].

### 1.3. Internalized Homophobia

SMM of color can experience a lack of family support due to rejection, which may result in both depression and internalized homophobia [25]. Internalized homophobia is an individual’s internal conflict with their sexual identity and creates negative perceptions that are directed internally [25]. This can have deleterious effects on overall mental health and well-being [26,27,28,29] resulting from the interconnectedness of sexual identity, family factors, and internalized homophobia on depression. In addition, some research has found that racism compounded with internalized homophobia has been associated with maladaptive outcomes on sexual minorities [30,31]. Several researchers have framed internalized homophobia as an aspect of the coming-out process that is marked by low self-esteem and heightened levels of depression and anxiety [26,32,33]. Longitudinal research has highlighted that the lessening of internalized homophobia over time is associated with positive health outcomes among SMM [33].

### 1.4. Stressful Life Events

Another factor to consider related to depression in SMM is stressful life events. SMM commonly experience stigma and discrimination during their lifetime [34]. These experiences are ongoing and experienced across multiple everyday domains of livelihood, such as education, employment, housing, recreation, and through religious worship [35]. Verbal, physical, and sexual violence are also commonly experienced [34,35]. Moreover, prior research has indicated that SMM experience multiple layers of stigmas daily such as racism, racial and social discrimination, and internalized heterosexism, which negatively impacts their mental health [31]. In addition, SMM are also more likely to avoid accessing healthcare treatment if they believe the healthcare clinicians will discriminate against them in case they are diagnosed with HIV or other sexually transmitted infections [36]. The interconnectedness of compounding effects from multiple forms of stigma and discrimination is overly burdensome and can lead to depression in this population [37].

### 1.5. The Present Study

Building on prior research, the authors contend that family-based approaches that acknowledge the intersections of sexual identity, race, family factors, stressful life events, and occurrences of internalized homophobia are critical for improving SMM’s mental health and related outcomes [38,39]. This has been recommended for use in quantitative analyses to explain variance and further understand the multi-contextual, holistic, social identities of individuals in health outcomes research [5,38,39]. The purpose of this study was to use an intersectional methodological approach to examine the relationship of family factors, internalized homophobia, and stressful life events on depression among SMM of color. We hypothesize that a lack of family support, internalized homophobia, and stressful life events are positively correlated with depression among SMM men of color.

## 2. Methods

### 2.1. Procedures

The Healthy Young Men’s (HYM) Cohort Study is a longitudinal study (baseline, 6-month follow-up assessments) conducted with 448 Black, Latino, or multi-racial/ethnic (i.e., they identify in part as Black and/or Latino) YMSM. The study design described elsewhere [40,41,42,43], involved the recruitment of YMSM in Los Angeles, CA, USA, using both venue-based and social media recruitment strategies from May 2016 through September 2017. The HYM cohort study aims were to prevent and reduce HIV incidence among young SMM of color by examining the barriers and protective factors that contribute to their engagement in care. Young SMM who were HIV seronegative (*N* = 448) and those who were seropositive (*N* = 50) were eligible to participate in the study. General eligibility criteria were: (1) 16 to 24 years of age; (2) male assignment at birth; (3) self-identify as gay, bisexual, or uncertain about their sexual orientation; (4) report having a sexual experience with a man within the last 12 months; (5) self-identify as African American/Black, Hispanic/Latino, or multiracial ethnic; and (6) reside in Los Angeles or a surrounding county with no plans on moving outside of the area for at least six months. The current study uses data from wave 5 of Latino and Black SMM (*N* = 372).

The HYM primary study is described thoroughly elsewhere [40,41,42,43]. Institutional Review Board approval was obtained from Children’s Hospital Los Angeles.

### 2.2. Measures

The outcome variable for this study is depression. Depression was measured using the Brief Symptom Inventory (BSI-18) [44], which assessed current symptoms of somatization, depression, and anxiety (i.e., within the past seven days). The BSI has been used with this particular population. To identify participants whose symptoms reached a clinical level of concern, BSI subscale scores were converted to T scores. Scores greater than or equal to 63 indicate clinical concern. To compare participants above and below the threshold for clinical concern, scores for somatization, depression, and anxiety were dichotomized (<63, no clinical concern = 0, ≥63, clinical concern = 1) [45]. The alpha for the depression subscale was 0.84.

Several contextual variables were collected. Age was treated as a continuous variable. Employment status was measured (1 = full time, 2 = not working at this time and not looking for employment, 3 = not working at this time and looking for employment). Sexual identity options were (1 = gay, 2 = heterosexual, 3 = bisexual, 4 = other same sex, 5 = pansexual, 6 = unsure/question, 7 = other).

Several criteria were used to measure family context. Family social support [46] was measured using a four-item scale (ranging from 1 = very strongly disagree to 4 = agree) asking the respondents to agree or disagree with the following: (1) “My family tries to help”; (2) “I can talk about my problems with my family”; (3) “My family is willing to help me make decisions”; and (4) “I get the emotional help and support I need from family”. FSS scores were created by averages across item responses (α = 0.91) and standardized before analysis. Separation from family and friends because of sexuality (SFFS) was measured using a single item—“As an adult, how often have you had to move away from friends or family because of your sexuality?”—from a subscale of homophobic experiences, with scores ranging from a single instance (1) to many times (4). Perceived embarrassment and hurt due to sexuality by family (PEHS) was measured using a single item—“Today, how often do you feel that your sexuality hurts and embarrasses your family?”—from a subscale of homophobic experiences, with scores ranging from never (1) to many times (4) [46].

The variable stressful life events was measured by participants indicating whether they had experienced each of the 32 stressful life events during the last six months (0 = no, 1 = yes). The list of stressful life events included a subset of 27 items from Nott and Vedhara’s [47] scale for stressful events among men who have sex with men living with HIV (e.g., “You had problems/difficulties with a close friend”) and a further five items representing additional stressful events identified as salient to homosexual young men in prior research conducted by this team (“You came out to your family”) [47]. A score representing the total number of stressful life events was created by summing the number of events endorsed. In addition, for each stressful life event endorsed, participants rated the severity of that stressor over the last 6 months (1 = low, 10 = high) [41,43].

*Internalized homophobia* was measured using the Revised Internalized Homophobia Scale (IHP-R); [40,41] it included four items measured on a scale ranging from strongly disagree (0) to strongly agree (3). IHP scores were created by averaging across item responses (α = 0.89).

### 2.3. Data Analysis

All analyses were conducted on observations that included non-missing data for the outcome, depression. Statistical tests of association were conducted between the measures described in the methods and the continuous outcome, depression. Table 1 provides descriptive statistics for the study sample. Table 2 provides bivariate correlations, mean, and standard deviations among independent variables: family social support, stressful life events, internalized homophobia, SFFS, and PEHS; the control variables are: age, sexual identity, and employment status on the outcome depression. Next, a multiple regression was conducted on the independent variables and the dependent variables (Table 3) (*N* = 372). Post-estimation tests were performed after the analysis to check for multicollinearity between variables. Survey data were analyzed using listwise deletion. For survey scales, a mean score of the scale items was generated for participants with non-missing data. All analyses were conducted in STATA 16.

## 3. Results

### 3.1. Descriptive Statistics

Table 1 provides descriptive information on mean scores for major study variables from wave 5. The analytic sample consisted of 372 participants between the ages of 18 and 29; the mean age was 24; range = 18–29. Fifty-eight percent self-identified as Latinx (*N* = 244), 23% as African American (*N* = 87) and 22% as mixed race (*N* = 83). The majority of the sample (78%) self-identified as homosexual, 11% being bisexual, 4% MSM, 3% pansexual, 2% heterosexual, and approximately 2% unsure or questioning. In addition, most of the sample reported working full-time (48%); 32% self-reported working part-time. Fifteen percent of the sample reported not working but looking for work, and 5% reported not working and not looking for work.

Overall, participants did not experience clinical levels of depression (*M* = 49, *SD* = 9.61). Generally, 13% had experienced some form of a stressful life event and 38% of the sample struggled with internalized homophobia. Moreover, 39% of the sample reported they were separated from their family and friends because of their sexuality, and 30% reported feeling embarrassed and hurt due to sexuality by family. Lastly, 71% reported they had received some social support from their family.

### 3.2. Bivariate Correlation Analysis

Table 2 provides bivariate correlations between the primary study variables and the outcome variable of depression symptoms. The results of the Pearson correlation indicated that there was a significant positive association between stressful life events and depression symptoms (*r* = 0.30, *p* < 0.001). There were positive relationships between internalized homophobia and depression (*r* = 0.29, *p* < 0.001) and stressful life events (*r* = 0.14, *p* < 0.05). A negative correlation existed between family social support and depression (*r* = −0.25, *p* < 0.001) serving as a buffer, stressful life events (*r* = −0.15, *p* < 0.05), and internalized homophobia (*r* = 0.10, *p* < 0.05). The results also indicated a positive correlation between SFFS and depression symptoms (*r* = 0.28, *p* < 0.001), stressful life events (*r* = 0.30, *p* < 0.01), internalized homophobia (*r* = 0.24, *p* < 0.001), and family support (*r* = −0.19, *p* = 0.002). PEHS was positively associated with depression (*r* = 0.19, *p* < 0.001), as well as stressful life events (*r* = 0.24, *p* < 0.001), family social support (*r* = 0.15, *p* < 0.001), and age (*r* = 0.35, *p* < 0.001), and there was a negative relationship between depression and SFFS (*r* = −0.20, *p* < 0.001). Employment was positively correlated with depression (*r* = 0.15, *p* < 0.05), stress (*r* = 0.10, *p* < 0.05), internalized homophobia (*r* = 0.08, *p* < 0.05), and social discrimination (family values) *(r* = 0.12, *p* < 0.05), and negatively associated with family support (*r* = −0.13, *p* < 0.010).

### 3.3. Multiple Regression

The overall model (Table 3) was statistically significant, and the variables accounted for 24% of the amount of variance in depression, R^2^ = 0.24, F (16, 355) = 6.96, *p* < 0.001. Stressful life events were found to be statistically significant and positively associated with depression (ß = 0.18, *p* = 0.026). Findings indicated that an 86% increase in family social support was associated with a −0.14 decrease in depression (ß = −0.14, *p* = 0.004), while holding all other variables constant. For every one-unit increase in internalized homophobia, there was a 0.21 increase in depression (ß = 0.21, *p* < 0.001). PEHS was statistically significant and positively associated with depression (ß = 0.09, *p* < 0.001). Employment (not working and looking for work) was positively associated with depression among SMM (ß = 0.13, *p =* 0.12). Lastly, identifying as pansexual was positively associated with depression for SMM (ß =2.61, *p* = 0.05).

## 4. Discussion

The purpose of this secondary analysis was to examine how the presence or lack of family support as well as internalized homophobia influenced depression rates among SMM. This study utilized intersectionality theories, which included both risk and protective processes, and an analytical model to investigate depression among SMM. The significant association between family social support and SMM who reported stressful life events, depression, and internalized homophobia is consistent with research that indicated internalized homophobia may cause stigma anticipation and perceptions of negative treatment by their parents based on their identities [27,48]. Participants in this study did not report moderate or high rates of depression. This may be due to SMM being less likely than other groups to report depression [49].

Our study findings indicated that an increase in family social support was associated with less depression, which is consistent with previous studies that family support serves as a protective mechanism against negative health outcomes [16,17,50]. Black and Latinx SMM in this study may have supportive networks of family members who support them during stressful and joyful times. In addition, they may also have positive relationships within these networks including their parents. These relationships between SMM of color and their families may have a positive influence on their overall mental health. 

In addition, we found significant negative associations between stressful life events, internalized homophobia, perceived embarrassment and hurt due to sexuality by family, and depression. The primary implication of this finding is that more effort is needed to develop networks of support in Black and Latinx communities centered on parents and families, including fictive kin—individuals who may not be blood relatives—to reduce depression and promote mental health and well-being in this population. This highlights the importance of the family and members of the social network who are considered family because of their unconditional support and friendship.

This study contributes to the literature as our findings support the belief that the influence from the microsystem is salient to modifying mental health outcomes [49]. The indirect influence from mesosystems and categories of identity on depression in Black and Latinx SMM suggests a positive relationship between family values and depression as well as between family embarrassment and depression. Previous studies indicated that discriminatory behavior and/or treatment by parents could increase feelings of stigma along with internalized homophobia, which could further marginalize SMM in terms of their feelings of oppression and the toll this may take on them [49,50]. Further, homophobia and discrimination in African American communities tend to create environments that are less friendly to sexual minorities. This delays sexual orientation disclosure and increases the risk of contracting HIV [51,52,53,54].

## 5. Limitations

The results of our study should be interpreted considering several limitations. First, this was a cross-sectional sub-study, and our analysis only captures one point in time (wave 5) and does not account for longitudinal or temporal changes. Due to this, the role of the ecodevelopmental or intersectional processes associated with depressive symptoms was not assessed over time. In addition, our findings may not be generalizable to all youth and young adults of color in the United States. Further, our findings are limited to a sample of young SMM from Los Angeles, there is a lack of a random sample, and the parent study includes the mental element of the ecodevelopmental theory, so no causal or directional inferences were drawn, though there could be unknown threats to internal validity, including maturation and history. Moreover, maturation noted the possibility that mental or physical changes happening to the participant beyond the researcher’s control might have affected the participants’ responses.

## 6. Implications and Conclusions

Our findings suggest the necessity of using an intersectional lens to examine the relationship between SMMs’ mental health and parent/family social support. This is particularly important considering the lack of research on the intersecting identities of SMM. More research examining the intersections of family and culture is needed due to the high prevalence of depression among this population compared to their peers. The results from our study prompt the need for research and health practitioners to better navigate uncharted territory in relationships between family and young SMM to promote the use of specific interventions to improve health outcomes in this group.

## Figures and Tables

**Table 1:** Demographics (*N* = 414).

Variable	Mean	SD	Range
Depression	49.06	9.61	41–93
Stressful Life Events	5.11	4.47	0–38
Internalized Homophobia	6.25	2.88	4–16
SFFS	1.22	0.59	1–4
PEHS	2.00	0.93	1–4
Family Social Support	5.00	1.49	1–7
Age	24.00	1.00(SE)	18–29

SFFS, separation by family and friends because of sexuality; PEHS, perceived embarrassment and hurt due to sexuality by family.

**Table 2:** Correlations on independent variables on depression.

Depression	1	2	3	4	5	6	7	
Stressful Life Events	0.31 ***	1						
Internalized Homophobia	0.29 ***	0.14 *	1					
Family Support	−0.25 ***	−0.15 *	−0.010 *	1				
SFFS	0.28 ***	0.30 ***	0.24 ***	−0.19 ***	1			
PEHS	0.19 ***	0.24 ***	0.15 *	−0.20 ***	0.35 ***	1		
Age	−0.06	0.01	0.01	0.01	0.01	−0.03	1	
Employment	0.15 *	0.10 *	0.08 *	−0.13 **	0.12 *	−0.0264	0.0427	1

* *p* < 0.05, ** *p* < 0.01, *** *p* < 0.001; SFFS, separation by family and friends because of sexuality; PEHS, perceived embarrassment and hurt due to sexuality by family.

**Table 3:** Multiple regression on depression (N = 372).

Variable	B	SE	ß
Depression			
Stressful Life Events	0.16 ***	0.05	0.18
Internalized Homophobia	1.23 ***	0.30	0.21
Family Social Support	−0.41 **	0.14	−0.14
SFFS	0.55 *	0.24	0.09
PEHS	0.29	0.37	0.04
Age	−0.12	0.10	−0.06
Sexual Identity			
Heterosexual (straight)	−2.02	1.97	−0.05
Bisexual	−0.44	0.64	−0.03
Other same sex (e.g., Man who has s..)	0.95	0.99	0.05
Pansexual	2.61 *	1.28	0.10
Unsure/questioning	−0.64	2.23	−0.01
Other—Please specify:	−2.03	2.22	−0.04
Employment			
Yes, working full-time	−0.61	0.45	−0.07
Not working at this time and NOT looking	−0.88	0.99	−0.04
Not working at this time and looking	1.52 *	0.64	0.13

* *p* < 0.05, ** *p* < 0.01, *** *p* < 0.001; SFFS, separation by family and friends because of sexuality; PEHS, perceived embarrassment and hurt due to sexuality by family.

## Data Availability

The data is not available to the public without the consent of the PI.
